# Association between plasmatic oxidative stress and thrombosis in primary antiphospholipid syndrome

**DOI:** 10.1007/s11239-021-02509-0

**Published:** 2021-07-05

**Authors:** Camila O. Vaz, Bruna M Mazetto, Pedro Eduardo N. S. Vasconcelos, Larissa B. Bastos, Maria Aparecida Cursino, Júlia Coelho França Quintanilha, Gabriela Lisiane Tripiquia Vechiatto Mesquita, Ana Paula R. Santos, Bruna Cardoso Jacintho, José Diogo Oliveira, Joyce Annichino-Bizzachi, Fernanda A. Orsi

**Affiliations:** 1 Department of Pharmacology, School of Medical Sciences, University of Campinas, Campinas, SP Brazil; 2 School of Medical Sciences, University of Campinas, Campinas, SP Brazil; 3 School of Pharmaceutical Sciences, University of Campinas, Campinas, SP Brazil; 4 Department of Medical Sciences, School of Medical Sciences, University of Campinas, Campinas, SP Brazil; 5 Department of Clinical Medicine, School of Medical Sciences, University of Campinas, Campinas, SP Brazil; 6 Hematology and Hemotherapy, Center University of Campinas, Campinas, SP Brazil; 7 Department of Clinical Pathology, School of Medical Sciences, University of Campinas, Campinas R. Tessália Vieira de Camargo, 126. Cidade Universitária, Campinas, SP 13083-887 Brazil; 8 Leiden University Medical Center (LUMC), Leiden, The Netherlands

**Keywords:** Antiphospholipid syndrome, Plasmatic oxidative stress, Thrombosis, Oxidized biomolecules

## Abstract

Antiphospholipid antibodies induce a pro-inflammatory and hypercoagulable state that lead to increased risk of thrombosis. Whether oxidative damage contributes thrombosis risk is a matter of debate. We evaluated the association between oxidative stress and thrombosis in primary antiphospholipid syndrome (t-PAPS). Plasma total antioxidant capacity and the levels of malondialdehyde (TBARs), carbonyl protein, and 8-isoprostane in plasma were determined in a group of patients with t-PAPS and in individuals without a history of thrombosis (controls) using commercial ELISA assays. The levels of these plasma markers of oxidative stress were compared between t-PAPS and controls using Mann–Whitney test. A total of 70 patients with t-PAPS and 74 controls were included. Overall, measurements of all plasma oxidative stress markers were similar between t-PAPS patients and controls. In a subgroup analysis, patients with t-PAPS and arterial thrombosis had a higher antioxidant capacity as compared to controls. Thrombotic PAPS was not associated with increased levels of oxidative stress markers, in comparison with individuals without thrombosis. Even though it is not possible to rule out that a mild oxidative damage, not detected by plasma markers, occurs in t-PAPS, our results suggest that measuring plasma oxidative stress markers has limited clinical relevance in t-PAPS.

## Highlights

- In vitro studies demonstrated that antiphospholipid antibodies could trigger a pro-oxidative state.
- The clinical relevance of oxidative damage for thrombosis risk in primary antiphospholipid syndrome is not determined.
- In this study, plasma levels of oxidative stress markers were not associated with thrombosis in antiphospholipid syndrome.
- Plasma oxidative stress markers have limited role as biomarkers of thrombosis in antiphospholipid syndrome.
- Biomarkers capable of determining the risk of thrombosis in individuals with antiphospholipid antibodies are still needed.

## Background

Antiphospholipid syndrome (APS) is a chronic disorder characterized by thromboembolic events or obstetric complications combined with the presence of at least one antiphospholipid antibody (aPL), such a lupus anticoagulant (LAC), anticardiolipin (aCL) and anti-beta2-glycoprotein 1 (aβ2GP1) [1]. The thrombotic manifestations are venous, arterial or microvascular thrombosis. APS is diagnosed in approximately 10% of young adults with a stroke and 20% of patients with unprovoked venous thrombosis [2, 3]. Knowledge about the pathological mechanisms at the basis of APS-associated hypercoagulability and thrombosis is yet evolving and. Recently, the presence of aPL has been associated with a pro-oxidative state, which may be a result of the reduction of antioxidant mechanisms [4–6].

In vitro studies demonstrated that aPLs can deplete antioxidant mechanisms, and trigger a pro-oxidative state by increasing superoxide dismutase expression in leucocytes, which provokes mitochondrial overload and unbalance in ROS production [7]. Furthermore, aPLs was shown capable of increasing the activity of nitric oxide synthase induced (i-NOS) enzyme and the production of peroxynitrites, which are very reactive molecules that can trigger oxidative reactions [8].

Despite being biologically plausible, the above-cited oxidative mechanisms have not yet been proven clinically relevant in patients with PAPS. Therefore, this study was aimed to evaluate whether plasma markers of oxidative stress are associated with thrombosis in PAPS.

## Methods

### Participants’ selection

The study participants were enrolled between May 2018 and July 2019. Consecutive patients with PAPS and thrombosis treated at the Hematology and Hemotherapy Center of the University of Campinas were included in the study. Inclusion criteria for patients comprised the diagnosis of PAPS and the history of at least one thrombotic episode confirmed by an imaging exam. PAPS was diagnosed in patients with a persistently positive aPL antibodies; which was defined as persistent positive LAC; persistent positive IgG or IgM aCL at moderate to high titles (> 40 GPL or MPL) or persistent positive (> the 99th percentile) IgG/IgM aβ2GP1, on two occasions, with an interval of at least 12 weeks. aPL assays were performed according to the guidelines of the International Society of Thrombosis and Haemostasis (ISTH) and the Clinical and Laboratory Standard Institute (CLSI). Plasma samples were assayed by dilute Russell's viper venom time (dRVVT) and Silica Clotting Time (SCT) techniques for LAC detection. IgG or IgM aCL and IgG/IgM aβ2GP1 were confirmed using semi-quantitative “in house” ELISA immunological assays, with cardiolipin or β2GP1 as antigen (Sigma-Aldrich, USA), as previously described [9–11]. Patients with other systemic autoimmune diseases were classified as secondary APS and were excluded from the study. Clinical and demographic information of the patients were obtained from electronic medical records.

Individuals without a prior thrombosis, matched by the gender and age distribution with the patients, were included in the study as controls, these individuals were selected among university students, hospital staff and blood donors. Clinical and demographic information of controls without thrombosis were assessed by interviews. Patients and controls with systemic autoimmune diseases, neoplasia, cognitive, hearing, or speech impairments and pregnancy were excluded.

The study was approved by the Local Ethics Committee in Research (CAAE: 83102317.5.0000.5404). All participants provided and informed consent to participate in the study. By signing the informed consent form, all participants also agreed with the publication of their data as long as they remained anonymous.

### Plasma markers of oxidative stress

Three 3.5 mL tubes (two containing ethylenediaminetetraacetic acid [EDTA] and one containing 0.105 mol/L − 3.2% buffered sodium citrate) were used to collect whole blood from each of the participants. After collection, whole blood samples were centrifuged for ten minutes at − 4 °C and 2500 rpm for plasma separation. The plasma obtained was aliquoted and stored in a freezer at − 80 °C until the evaluation of plasma markers of oxidative stress.

The markers of oxidative stress used in this study comprised markers of total antioxidant capacity and markers of oxidative damage. To access plasma oxidative damage, we measured plasma levels of malondialdehyde (MDA), carbonylated proteins, and 8-isoprostanes. All markers were quantified by their commercial assay kits (Cayman Chemical®, Michigan, USA), according to the manufacturer’s instructions.

For the evaluation of the total antioxidant capacity, plasma samples from 51 patients and 54 controls were tested. Total antioxidant capacity assay measures the capacity of the enzymatic and molecular antioxidants in plasma to avoid oxidation of the chromogenic compound ABTS (2.2′-azino-di-[3-ethylbenzothiazoline-sulfonate) by metmyoglobin. High values of total antioxidant capacity may indicate higher protection against oxidative damage. MDA is a secondary organic product formed by the peroxidation of polyunsaturated fatty acids. The quantification of this biomarker is based on the reaction of the MDA contained in the sample with thiobarbituric acid. High levels of MDA in body fluids indicate increased oxidative damage. Likewise, increased levels of protein carbonyl indicate an oxidative imbalance, once carbonylation reactions in organic substrates are performed by ROS overexpression. 8-isoprostane consists of a eucosanoid produced by the non-enzymatic oxidation of arachidonic acid and its concentration in the biological sample is relative to free 8-isoprostane in plasma and directly proportional to the oxidative impairment.

### Statistical analysis

Descriptive analysis was expressed as frequency and percentage for categorical variables and point estimates (mean or median) and dispersion [standard deviation (SD) or interquartile range (IQR)] for the numerical variables. The comparison of numerical variables between two groups was performed using the unpaired Mann–Whitney test and between three or more groups the Kruskal Wallis test. The significance level adopted for this part of the study was 5% (p < 0.05). Data analysis was performed using SPSS Statistics for Windows, Version 20.0 (IBM Corp. Released 2011. IBM Armonk, NY: IBM Corp).

## Results

Seventy patients with t-PAPS and 74 controls were included in the study. Most of t-PAPS patients were women (64.3%) and the mean age was 41 (SD = 15) years. In the control group, the mean age was 41 (SD = 11) and most of the participants were women (60.8%), as well. The prevalence of kidney disease, hypertension, diabetes and dyslipidemia was higher in t-PAPS than in controls. Statins use, alcohol use and smoking were also more frequent among patients than among controls. Demographic and clinical characteristics of both groups are described in Table 1.

**Table 1:** Clinical and demographic characteristics of thrombotic- PAPS and controls

Characteristics	Controls (n = 74)	t-PAPS (n = 70)
Demographic data		
Age, mean (SD)	41 (11)	41 (15)
Sex n (%)		
Female	45 (60.8)	45 (64.3)
Ethnicity n (%)		
White	59 (79.7)	52 (74.3)
Black	7 (9.5)	7 (10.0)
Others	8 (10.8)	11 (15.7)
Education n (%)		
Primary education	1 (1.4)	23 (32.9)
Secondary education	4 (5.4)	40 (57.1)
Higher education	69 (93.2)	4 (5.7)
No formal education	0	3 (4.3)
Cardiovascular risk factors n (%)		
Hypertension	7 (9.5)	25 (35.7)
Diabetes	2 (2.7)	5 (7.1)
Chronic kidney disease	0	6 (8.6)
Dyslipidemia	8 (10.8)	27 (38.6)
Statins use	4 (5.4)	13 (18.6)
Smoking or alcohol abuse	19 (25.7)	28 (40.0)

*SD* standard deviation

t-PAPS was characterized mainly by non-provoked venous thromboembolism (VTE) or stroke, the patients’ mean age when the first thrombotic event occurred was 33.5 years old (SD = 13.7). A total of 21 patients (28.6%) had recurrent thrombotic events, with recurrences mostly occurring as VTE or stroke. Triple positivity for aPL was detected in 24.3% of the patients. All patients were using warfarin and the latest thrombotic event had occurred 64 months (SD = 58.1) prior to the inclusion in the study. Controls had no symptoms of thrombosis or prior history of thrombosis. They were not using any anticoagulant or antiplatelet agent when included in the study. All clinical and laboratory features related to t-PAPS presentation are demonstrated in Table 2.

**Table 2:** Clinical and laboratory features related to thrombotic episodes in PAPS patients

Clinical and laboratory features	PAPS patients (n = 70)
Number of thrombotic episodes, mean (SD)	1.4 (0.8)
Age in thrombotic episode, mean (SD)	33.5 (13.7)
Characteristics n (%)	
Provoked	22 (31.4)
Non-provoked	46 (65.7)
NA	2 (2.9)
Site of thrombotic episode n (%)	
Arterial thombosis	24 (34.3)
PAD	5 (7.1)
Stroke	19 (27.1)
Venous thrombosis	46 (65.7)
DVT	36 (51.4)
Unusual site	10 (14.3)
Multiple thrombosis n (%)	20 (28.6)
Site of recurrent thrombosis n (%)	
PAD	1 (1.4)
Stroke	6 (8.6)
DVT	10 (14.3)
Unusual site	3 (4.3)
Time elapsed since the last thrombosis in months, mean (SD)	64.6 (58.1)
Recurrent miscarriages, n (%) (out of the 35 women with at least one pregnancy)	16 (45.7)
aPL profile n (%)	
Lupus anticoagulant	59 (84.3)
Anticardiolipin antibody IgM	6 (8.6)
Anticardiolipin antibody IgG	22 (31.4)
Anti-β2-glycoprotein I antibody IgM/IgG	35 (50.0)
Triple positivity for aPL	17 (24.3)
Anticoagulant treatment n (%)	
Warfarin	70 (100)

*t-PAPS* thrombotic primary antiphospholipid syndrome, *SD* standard deviation, *NA* not available, *PAD* peripheral arterial disease, *DVT* deep vein thrombosis, *aPL* antiphospholipid antibodies

**Fig. 1:**
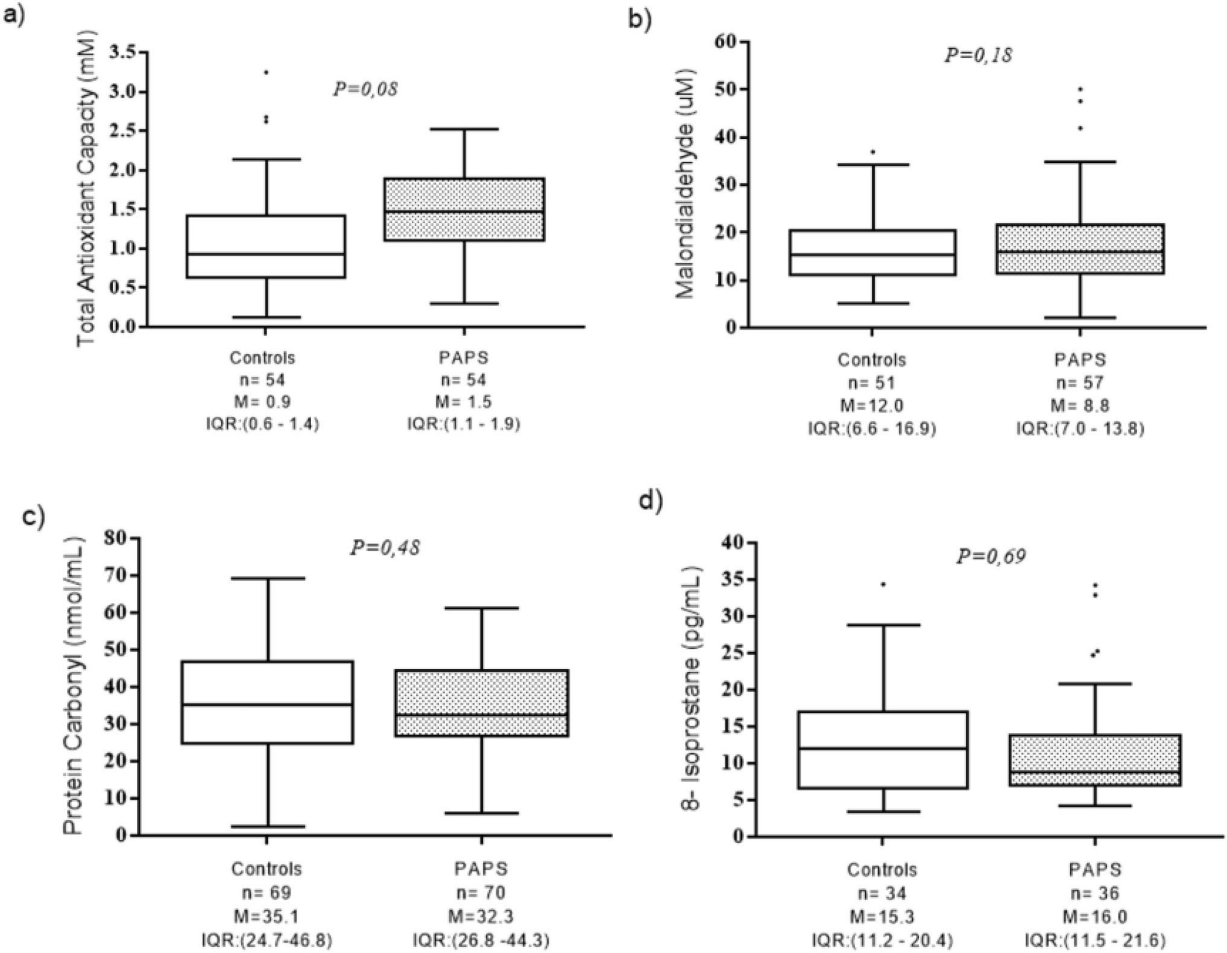
Levels of plasma oxidative stress markers in controls and thrombotic-PAPS patients. Boxplots represent the median and interquartile range of the results obtained by the quantitative tests of **a** Total antioxidant capacity; **b** Malondialdehyde; **c** Protein carbonyl; **d** 8-isoprostane. P values were calculated using Mann–Whitney test. *n* number of tested individuals, *M* median, *IQR* interquartile range, *P* P value, *PAPS* primary antiphospholipid syndrome

Results of the plasma markers of oxidative stress are shown in Fig. 1. The median value of total plasma antioxidant capacity was 1.05 mM (IQR 1.1–1.9) in t-PAPS patients and 0.9 mM (IQR 0.6–1.4) in controls (P = 0.08). The median value of MDA was 8.8 umol (IQR 7.0–13.8) in t-PAPS patients and 12.0 umol (IQR 6.6–16.9) in controls (P = 0.18). The plasma levels of carbonylated proteins were similar between patients (median 32.3 nmol/mL, IQR 26.8–44.3) and controls (median 35.1 nmol/mL, IQR 24.7–46.8; P = 0.48) in controls. The mean concentration of 8-isoprostanes was 16.0 pg/mL (IQR 11.5–21.6) in patients and 15.3 pg/mL (IQR 11.2–20.4) in controls (P = 0.69).

Next, t-PAPS patients were grouped according to the disease clinical manifestations, as follows: i. site of thrombosis (arterial or venous); ii. non-provoked and provoked thrombosis; iii. single and recurrent thrombosis and iv. triple positivity for the aPLs and non-triple positivity for the aPLs. The total antioxidant capacity was increased in the subgroup of patients with arterial thrombosis as compared to those with venous thrombosis and controls (p = 0.01). The results of the other markers of oxidative stress were similar between the subgroups.

We also grouped patients and controls according to the presence of morbidities related to increased cardiovascular risk, such as hypertension, dyslipidemia, and diabetes. No differences were observed in the levels of markers of oxidative stress between individuals with and without cardiovascular risk factors. Subgroups analysis are demonstrated in Table 3.

**Table 3:** Oxidative stress markers by subgroups of t-PAPS severity

	Total antioxidant capacity (mM)		MDA (umol)		Protein Carbonyl (nmol/mL)		8-isoprotanes (pg/mL)	
	n	median (IQR)	n	median (IQR)	n	median (IQR)	n	median (IQR)
Controls	54	0.9 (0.6–1.4)	51	12.0 (6.6–17.0)	69	35.1 (24.8–46.8)	34	15.3 (11.2–20.4)
Venous thrombosis	34	1.4 (1.0–1.8)	39	8.5 (7.0–12.8)	46	32.7 (27.7–45.1)	25	15.9 (10.7–21.5)
Arterial thrombosis	17	1.7 (1.2–2.2)	18	9.6 (7.2–14.4)	24	32.0 (22.4–42.1)	11	16.0 (11.5–18.0)
P value		0.01		0.39		0.57		0.91
Controls	54	0.9 (0.6–1.4)	51	12.0 (6.6–17.0)	69	35.1 (24.8–46.8)	34	15.3 (11.2–20.4)
Single thrombosis	37	1.4 (1.0–2.0)	39	8.6 (7.1–13.9)	50	33.7 (26.8–45.1)	24	16.1 (11.4–22.9)
Multiple thrombosis	14	1.5 (1.3–1.9	18	9.16 (6.6–13.2)	20	31.6 (23.5–37.9)	12	15.9 (10.7–17.8)
P value		0.16		0.41		0.66		0.3
Controls	54	0.9 (0.6–1.4)	51	12.0 (6.6–17.0)	69	35.1 (24.8–46.8)	34	15.3 (11.2–20.4)
Non-triple positive	40	1.6 (1.1–2.0)	44	8.9 (7.1–13.4)	53	33.2 (26.7–46.1)	27	15.8 (11.4–18.3)
Triple positive	11	1.2 (1.0–1.5)	13	8.3 (6.3–16.0)	17	30.4 (26.2–38.4)	9	19.3 (10.7–29.1)
P value		0.12		0.41		0.36		0.46

*t-PAPS* thrombotic primary antiphospholipid syndrome

P values were calculated by Kruskal Wallis

## Discussion

In this study, the plasma level of oxidative stress markers was similar between t-PAPS patients and controls. This finding was unexpected because previous studies have reported that aPL can trigger an oxidative damage. Plasma levels of paraxonase, which is an antioxidative enzyme linked to high-density lipoproteins (HDL), were associated with aPL titers in PAPS [12, 13]. In vitro studies showed that decreases in paraoxonase activity expose low-density lipoproteins (LDL) to an oxidative damage which ultimately leads to the formation of oxidized forms of LDL (oxLDL), a very reactive species capable of binding to β2GP1 and causing endothelial injury [14]. β2GP1, which is the main antigen target in APS, has also antioxidant activity and is related to the regulation of coagulation mechanisms, fibrinolysis, angiogenesis, and apoptosis [15]. When complexed with oxLDLs, β2GP1 loses its protective activity and becomes immunogenic by exposing cryptic antigens, which yields to the production of auto-antibodies [16]. Finally, antibodies directed against β2GP1 leads to the secretion of tissue factor (FT), growth factors, cytokines, and metalloproteases by endothelial cells, platelets, and monocytes [14, 16, 17]. Such pro-inflammatory state can cause a depletion of natural antioxidant mechanisms and susceptibility to damage generated by the action of ROS. MDA, carbonylated proteins and 8-isoprostanes, which are the biomarkers evaluated in this study, are produced as the result of lipids and proteins oxidation by the action of ROS.

From a clinical perspective, Stanisavljevic et al*.* [18] reported that patients with APS have an increase in lipid hydroperoxides and advanced protein oxidation products, which are peripheral markers of reactivity against lipids and proteins, when compared to healthy controls. Sciascia et al*.* [19] observed an increase in the concentration of 8-isoprostanes, another product from in vivo lipid oxidation, in patients with PAPS and SLE, mainly in those with triple positivity for aPLs.

Therefore, our findings were surprising because we expected to observe increased levels of oxidative plasmatic biomarkers in t-PAPS patients since previous studies have demonstrated that aPLs are capable of decreasing the activities of antioxidant enzymes [15, 17], and oxidative stress can contribute to the deregulation of the immune response and hypercoagulability in APS [7, 13, 20]. However, the discrepancies between our results and previous data can be explained by the fact that PAPS, unlike other autoimmune diseases such as SLE, is associated with a low-intensity inflammatory state, characterized by a mild increase in acute-phase inflammatory proteins and cells [21]. Therefore, it is possible that the mild inflammatory status of PAPS leads to a mild oxidative state [22], not detectable by plasma biomarkers.

With regard to the high total antioxidative capacity observed in t-PAPS patients with arterial thrombosis, it is possible that this phenomenon is due to the occurrence of reactive exacerbation of antioxidant defenses. The process of reactive exacerbation of antioxidant defenses consists of elevating endogenous antioxidant protection mechanisms to prevent tissue damage in situations of oxidative stress. This process has been described in smokers [23], asthmatic patients [24], after physical exercises in patients with metabolic and cardiopulmonary diseases [25] during coronary artery bypass surgery [26], and also during acute disease exacerbation chronic obstructive pulmonary disease [27]. In the context of SLE, there are controversial data regarding the measurement of patients' plasma antioxidant capacity, with studies reporting either increased or decreased antioxidant capacity in SLE [18, 28].

Our study has some limitations that must be highlighted. First, all included patients were already being treated with anticoagulants and it is not possible to exclude that these drugs affected the oxidative state in patients. Second, most thrombotic events occurred more than three years ago before the inclusion in the study, which could explain a mild pro-oxidative state. Third, we measured plasma markers of oxidative stress using indirect methods that may have been unable to access a mild oxidative status in these patients. Forth, the sample size is small which can be explained by the rarity of thrombotic PAPS.

In conclusion, this study demonstrated that t-PAPS was not associated either with the plasma levels of MDA, 8-isoprostane, carbonyl protein or with the plasma antioxidant capacity. Although the role of these plasma oxidative stress biomarkers seems to be limited in t-PAPS, it is not possible to exclude the possibility that a mild oxidative damage, not detected by these biomarkers, occurs in t-PAPS.

## References

[1] Hughes G (2007). Hughes syndrome: the antiphospholipid syndrome–a clinical overview. Clin Rev Allergy Immunol.

[2] Biggioggero M, Meroni PL (2010). The geoepidemiology of the antiphospholipid antibody syndrome. Autoimmun Rev.

[3] Meroni PL, Borghi MO, Raschi E, Tedesco F (2011). Pathogenesis of antiphospholipid syndrome: understanding the antibodies. Nat Rev Rheumatol.

[4] Cervera R, Piette JC, Font J, Khamashta MA, Shoenfeld Y, Camps MT, Jacobsen S, Lakos G, Tincani A, Kontopoulou-Griva I, Galeazzi M, Meroni PL, Derksen RH, de Groot PG, Gromnica-Ihle E, Baleva M, Mosca M, Bombardieri S, Houssiau F, Gris JC, Quere I, Hachulla E, Vasconcelos C, Roch B, Fernandez-Nebro A, Boffa MC, Hughes GR, Ingelmo M (2002). Antiphospholipid syndrome: clinical and immunologic manifestations and patterns of disease expression in a cohort of 1,000 patients. Arthritis Rheum.

[5] Ruiz-Irastorza G, Crowther M, Branch W, Khamashta MA (2010). Antiphospholipid syndrome. Lancet.

[6] Shi W, Chong BH, Chesterman CN (1993). Beta 2-glycoprotein I is a requirement for anticardiolipin antibodies binding to activated platelets: differences with lupus anticoagulants. Blood.

[7] Perez-Sanchez C, Ruiz-Limon P, Aguirre MA, Bertolaccini ML, Khamashta MA, Rodriguez-Ariza A, Segui P, Collantes-Estevez E, Barbarroja N, Khraiwesh H, Gonzalez-Reyes JA, Villalba JM, Velasco F, Cuadrado MJ, Lopez-Pedrera C (2012). Mitochondrial dysfunction in antiphospholipid syndrome: implications in the pathogenesis of the disease and effects of coenzyme Q(10) treatment. Blood.

[8] Alves JD, Grima B (2003). Oxidative stress in systemic lupus erythematosus and antiphospholipid syndrome: a gateway to atherosclerosis. Curr Rheumatol Rep.

[9] de Laat B, Derksen RH, Urbanus RT, de Groot PG (2005). IgG antibodies that recognize epitope Gly40-Arg43 in domain I of beta 2-glycoprotein I cause LAC, and their presence correlates strongly with thrombosis. Blood.

[10] Gharavi AE, Harris EN, Asherson RA, Hughes GR (1987). Anticardiolipin antibodies: isotype distribution and phospholipid specificity. Ann Rheum Dis.

[11] Montalvão S, Elídio PS, da Silva SS, de Moraes MB, Colella MP, de Paula EV, Appenzeller S, Annichino-Bizzacchi J, Orsi FA (2016). Clinical implications of the detection of antibodies directed against domain 1 of β2-glycoprotein 1 in thrombotic antiphospholipid syndrome. Thromb Res.

[12] Charakida M, Besler C, Batuca JR, Sangle S, Marques S, Sousa M, Wang G, Tousoulis D, Delgado Alves J, Loukogeorgakis SP, Mackworth-Young C, D'Cruz D, Luscher T, Landmesser U, Deanfield JE (2009). Vascular abnormalities, paraoxonase activity, and dysfunctional HDL in primary antiphospholipid syndrome. JAMA.

[13] Delgado Alves J, Mason LJ, Ames PR, Chen PP, Rauch J, Levine JS, Subang R, Isenberg DA (2005). Antiphospholipid antibodies are associated with enhanced oxidative stress, decreased plasma nitric oxide and paraoxonase activity in an experimental mouse model. Rheumatology.

[14] Lopez-Pedrera C, Barbarroja N, Jimenez-Gomez Y, Collantes-Estevez E, Aguirre MA, Cuadrado MJ (2016). Oxidative stress in the pathogenesis of atherothrombosis associated with anti-phospholipid syndrome and systemic lupus erythematosus: new therapeutic approaches. Rheumatology.

[15] Giannakopoulos B, Mirarabshahi P, Krilis SA (2011). New insights into the biology and pathobiology of beta2-glycoprotein I. Curr Rheumatol Rep.

[16] Passam FH, Giannakopoulos B, Mirarabshahi P, Krilis SA (2011). Molecular pathophysiology of the antiphospholipid syndrome: the role of oxidative post-translational modification of beta 2 glycoprotein I. J Thromb Haemost.

[17] Passam FH, Rahgozar S, Qi M, Raftery MJ, Wong JW, Tanaka K, Ioannou Y, Zhang JY, Gemmell R, Qi JC, Giannakopoulos B, Hughes WE, Hogg PJ, Krilis SA (2010). Beta 2 glycoprotein I is a substrate of thiol oxidoreductases. Blood.

[18] Stanisavljevic N, Stojanovich L, Marisavljevic D, Djokovic A, Dopsaj V, Kotur-Stevuljevic J, Martinovic J, Memon L, Radovanovic S, Todic B, Lisulov D (2016). Lipid peroxidation as risk factor for endothelial dysfunction in antiphospholipid syndrome patients. Clin Rheumatol.

[19] Sciascia S, Roccatello D, Bertero MT, Di Simone D, Cosseddu D, Vaccarino A, Bazzan M, Rossi D, Garcia-Fernandez C, Ceberio L, Stella S, Menegatti E, Baldovino S (2012). 8-isoprostane, prostaglandin E2, C-reactive protein and serum amyloid A as markers of inflammation and oxidative stress in antiphospholipid syndrome: a pilot study. Inflamm Res.

[20] Benhamou Y, Miranda S, Armengol G, Harouki N, Drouot L, Zahr N, Thuillez C, Boyer O, Levesque H, Joannides R, Richard V (2015). Infliximab improves endothelial dysfunction in a mouse model of antiphospholipid syndrome: Role of reduced oxidative stress. Vascul Pharmacol.

[21] Alvarez-Rodriguez L, Martinez-Taboada V, Calvo-Alen J, Beares I, Villa I, Lopez-Hoyos M (2019). Altered Th17/treg ratio in peripheral blood of systemic lupus erythematosus but not primary antiphospholipid syndrome. Front Immunol.

[22] Ames PR, Antinolfi I, Ciampa A, Batuca J, Scenna G, Lopez LR, Delgado Alves J, Iannaccone L, Matsuura E (2008). Primary antiphospholipid syndrome: a low-grade auto-inflammatory disease?. Rheumatology.

[23] Hackett NR, Heguy A, Harvey BG, O'Connor TP, Luettich K, Flieder DB, Kaplan R, Crystal RG (2003). Variability of antioxidant-related gene expression in the airway epithelium of cigarette smokers. Am J Respir Cell Mol Biol.

[24] Comhair SA, Bhathena PR, Farver C, Thunnissen FB, Erzurum SC (2001). Extracellular glutathione peroxidase induction in asthmatic lungs: evidence for redox regulation of expression in human airway epithelial cells. FASEB J.

[25] Fisher-Wellman K, Bell HK, Bloomer RJ (2009). Oxidative stress and antioxidant defense mechanisms linked to exercise during cardiopulmonary and metabolic disorders. Oxid Med Cell Longev.

[26] Luyten CR, van Overveld FJ, De Backer LA, Sadowska AM, Rodrigus IE, De Hert SG, De Backer WA (2005). Antioxidant defence during cardiopulmonary bypass surgery. Eur J Cardio-Thorac Surg.

[27] Sadowska AM, Luyten C, Vints AM, Verbraecken J, Van Ranst D, De Backer WA (2006). Systemic antioxidant defences during acute exacerbation of chronic obstructive pulmonary disease. Respirology.

[28] Firuzi O, Fuksa L, Spadaro C, Bousova I, Riccieri V, Spadaro A, Petrucci R, Marrosu G, Saso L (2006). Oxidative stress parameters in different systemic rheumatic diseases. J Pharm Pharmacol.

